# The Global Drug Facility as an intervention in the market for tuberculosis drugs

**DOI:** 10.2471/BLT.14.147256

**Published:** 2015-03-03

**Authors:** Nimalan Arinaminpathy, Thierry Cordier-Lassalle, Kaspars Lunte, Christopher Dye

**Affiliations:** a Department of Infectious Disease Dynamics, Imperial College London, St Mary’s Hospital, Praed Street, London W21PG, England.; b World Health Organization, Geneva, Switzerland.; c Stop TB Partnership, Geneva, Switzerland.; d Office of the Director-General, World Health Organization, Geneva, Switzerland.

## Abstract

**Objective:**

To investigate funding for the Global Drug Facility since 2001 and to analyse the facility’s influence on the price of high-quality tuberculosis drugs.

**Methods:**

Data on the price of tuberculosis drugs were obtained from the Global Drug Facility for 2001 to 2012 and, for the private sector in 15 countries, from IMS Health for 2002 to 2012. Data on funding of the facility were also collected.

**Findings:**

Quality-assured tuberculosis drugs supplied by the Global Drug Facility were generally priced lower than drugs purchased in the private sector. In 2012, just three manufacturers accounted for 29.9 million United Stated dollars (US$) of US$ 44.5 million by value of first-line drugs supplied. The Global Fund to Fight AIDS, Tuberculosis and Malaria provided 73% (US$ 32.5 million of US$ 44.5 million) and 89% (US$ 57.8 million of US $65.2 million) of funds for first- and second-line drugs, respectively. Between 2010 and 2012, the facility’s market share of second-line tuberculosis drugs increased from 26.1% to 42.9%, while prices decreased by as much as 24% (from US$ 1231 to US$ 939). Conversely, the facility’s market share of first-line drugs fell from 37.2% to 19.2% during this time, while prices increased from US$ 9.53 to US$ 10.2.

**Conclusion:**

The price of tuberculosis drugs supplied through the facility was generally less than that on the private market. However, to realize its full potential and meet the needs of more tuberculosis patients, the facility requires more diverse and stable public funding and greater flexibility to participate in the private market.

## ملخص

**الغرض:**

تحري تمويل مرفق الأدوية العالمي منذ عام 2001 وتحليل تأثير المرفق على أسعار أدوية السل عالية الجودة.

**الطريقة:**

تم الحصول على بيانات عن أسعار أدوية السل من مرفق الأدوية العالمي للفترة من 2001 إلى 2012، وبالنسبة للقطاع الخاص في 15 بلداً، من IMS Health للفترة من 2002 إلى 2012. وتم كذلك جمع البيانات حول تمويل المرفق.

**النتائج:**

تم تسعير أدوية السل مضمونة الجودة التي يقوم بتوريدها مرفق الأدوية بشكل عام بسعر أقل من الأدوية التي يتم شراؤها في القطاع الخاص. وفي عام 2012، بلغت مبيعات ثلاثة صانعين فقط 29.9 مليون دولار أمريكي من أصل 44.5 مليون دولار أمريكي بحسب قيمة أدوية الخط الأول التي تم توريدها. وقدم الصندوق العالمي لمكافحة الإيدز والسل والملاريا 73 % (32.5 مليون دولار أمريكي من أصل 44.5 مليون دولار أمريكي) و89 % (57.8 مليون دولار أمريكي من أصل 65.2 مليون دولار أمريكي) من الأموال إلى أدوية الخط الأول والخط الثاني، على التوالي. وبين عامي 2010 و2012، ازدادت حصة السوق من أدوية السل الخاصة بالمرفق التي تنتمي إلى الخط الثاني بنسبة تراوحت من 26.1 % إلى 42.9 %، في حين انخفضت الأسعار بنسبة كبيرة بلغت 24 % (من 1231 دولاراً أمريكياً إلى 939 دولاراً أمريكياً). وفي مقابل ذلك، انخفضت حصة السوق الخاصة بالمرفق من أدوية الخط الأول من 37.2 % إلى 19.2 % خلال هذه الفترة، في حين ازدادت الأسعار من 9.53 دولاراً أمريكياً إلى 10.2 دولاراً أمريكياً.

**الاستنتاج:**

كانت أسعار أدوية السل التي تم توريدها من خلال المرفق أقل عموماً من الأسعار في سوق القطاع الخاص. إلا أنه لتحقيق إمكاناته الكاملة وتلبية احتياجات المزيد من مرضى السل، يتطلب المرفق تمويلاً عمومياً أكثر تنوعاً وثباتاً ومرونة أكبر للمشاركة في سوق القطاع الخاص.

## 摘要

**目的:**

调查2001年以来全球药物机构（Global Drug Facility）的资金情况，分析该机构对高质量结核病药物价格的影响。

**方法:**

从全球药物机构收集2001至2012年15个国家的私营部门结核病药物的价格数据，从艾美仕市场调研咨询公司（IMSHealth）收集2002至2012年的此类数据，还收集有关该机构的资金的数据。

**结果:**

全球药物机构提供的有质量保证的结核病药物价格一般低于在私营部门购买的药物。在2012年，按照提供的一线药物价值计算，三家制药商就占到了4450万美元总额中的2990万美元。全球抗击艾滋病、结核病和疟疾基金分别为一线和二线药物提供73%（4450万美元中的3250万美元）和89%（6520万美元中的5780万美元）的资金。在2010年至2012年间，该机构二线结核病药物市场份额从26.1%提高到42.9%，而价格下降多达24%（从1231美元降至939美元）。相反，该机构在此期间一线药物的市场份额从37.2%降至19.2%，而价格从9.53美元提高到10.2美元。

**结论:**

全球药物机构提供的结核病药物的价格普遍低于私营市场的价格。然而，要充分发挥其潜力，满足更多肺结核患者的需要，该机构需要更加多元化和稳定的公共资金和更灵活地参与私营市场。

## Résumé

**Objectif:**

Examiner le financement du Dispositif mondial d'approvisionnement en médicaments depuis 2001 et analyser l'influence du Dispositif sur le prix des médicaments antituberculeux de haute qualité.

**Méthodes:**

Les données sur le prix des médicaments antituberculeux ont été obtenues auprès du Dispositif mondial d'approvisionnement en médicaments pour la période allant de 2001 à 2012 et pour le secteur privé dans 15 pays auprès d'IMS Health pour la période allant de 2002 à 2012. Des données sur le financement du Dispositif ont également été recueillies.

**Résultats:**

Le prix des médicaments antituberculeux de qualité garantie fournis par le Dispositif mondial d'approvisionnement en médicaments était généralement inférieur au prix des médicaments achetés dans le secteur privé. En 2012, 3 fabricants ont représenté à eux seuls 29,9 millions de dollars des États-Unis d'Amérique ($US) des 44,5 millions $US en valeur des médicaments de première intention fournis. Le Fond mondial de lutte contre le SIDA, la tuberculose et le paludisme a fourni 73% (32,5 millions $US sur les 44,5 millions $US) et 89% (57,8 million $US sur les 65,2 millions $US) des fonds pour les médicaments de première intention et de deuxième intention, respectivement. Entre 2010 et 2012, la part de marché du Dispositif pour les médicaments antituberculeux de deuxième intention a augmenté de 26,1% à 42,9% alors que les prix ont diminué de 24% (de 1231 $US à 939 $US). Inversement, la part de marché du Dispositif pour les médicaments de première intention a baissé de 37,2% à 19,2%, pendant que les prix ont augmenté de 9,53 $US à 10,2 $US.

**Conclusion:**

Le prix des médicaments antituberculeux fournis par le Dispositif était généralement inférieur à leur prix sur le marché privé. Cependant, pour exploiter pleinement son potentiel et répondre aux besoins de plus de patients tuberculeux, le Dispositif a besoin d'un financement public plus stable et plus varié et d'une plus grande flexibilité afin de participer au marché privé.

## Резюме

**Цель:**

Изучить финансирование, выделяемое на Глобальный механизм по обеспечению лекарственными средствами с 2001 года, и проанализировать влияние Механизма на стоимость высококачественных лекарственных препаратов для лечения туберкулеза.

**Методы:**

Были получены данные о стоимости противотуберкулезных лекарственных препаратов за период с 2001 по 2012 год, представленные Глобальным механизмом по обеспечению лекарственными средствами, а также данные для частного сектора в 15 странах за период с 2002 по 2012 год, представленные компанией IMS Health. Кроме того, были собраны данные о финансировании Механизма.

**Результаты:**

Стоимость противотуберкулезных лекарственных препаратов с гарантированным качеством, поставляемых посредством Глобального механизма по обеспечению лекарственными средствами, в целом была ниже стоимости лекарственных препаратов, приобретаемых в частном секторе. В 2012 году только три производителя отчитались о 29,9 миллионах долларов США из стоимости поставленных лекарственных препаратов первой линии, составившей 44,5 миллиона долларов США. Глобальный фонд по борьбе со СПИДом, туберкулезом и малярией выделил средства на лекарственные препараты первой и второй линии в объеме 73% (32,5 из 44,5 миллиона долларов США) и 89% (57,8 из 65,2 миллиона долларов США) соответственно. За период с 2010 по 2012 год доля Механизма на рынке противотуберкулезных лекарственных препаратов второй линии увеличилась с 26,1% до 42,9%, в то время как их стоимость снизилась на 24% (с 1 231 до 939 долларов США). Рыночная доля Механизма на рынке противотуберкулезных лекарственных препаратов первой линии, напротив, сократилась за этот период с 37,2% до 19,2%, в то время как их стоимость увеличилась с 9,53 до 10,2 долларов США.

**Вывод:**

Стоимость противотуберкулезных лекарственных препаратов, поставляемых посредством Механизма, в целом была ниже стоимости лекарственных препаратов в частном секторе рынка. Тем не менее, для реализации всего потенциала Механизма и удовлетворения потребностей большего числа пациентов с туберкулезом Механизму требуется более разноплановое и стабильное бюджетное финансирование и бóльшая гибкость для функционирования в частном секторе рынка.

## Resumen

**Objetivo:**

Investigar la financiación del Servicio Farmacéutico Mundial desde 2001 y analizar la influencia del Servicio en el precio de los medicamentos contra la tuberculosis de alta calidad.

**Métodos:**

Los datos sobre el precio de los medicamentos contra la tuberculosis se obtuvieron del Servicio Farmacéutico Mundial para el periodo comprendido entre 2001 y 2012, y de IMS Health para el sector privado en 15 países del año 2002 al año 2012. También se recogieron datos sobre la financiación del Servicio.

**Resultados:**

Por lo general, los medicamentos contra la tuberculosis con garantía de calidad suministrados por el Servicio Farmacéutico Mundial tenían un precio inferior que los medicamentos comprados en el sector privado. En 2012, tan solo tres fabricantes representaron 29,9 millones de dólares americanos (USD) de los 44,5 millones USD por el valor de los medicamentos de primera línea suministrados. El Fondo Mundial de Lucha contra el SIDA, la tuberculosis y la malaria proporcionó el 73 % (32,5 de los 44,5 millones de dólares americanos) y el 89 % (57,8 de 65,2 millones de dólares americanos) de los fondos para medicamentos de primera y segunda línea, respectivamente. Entre 2010 y 2012, la cuota de mercado del Servicio de medicamentos contra la tuberculosis de segunda línea aumentó del 26,1 % al 42,9 %, mientras que los precios disminuyeron en hasta un 24 % (de 1231 USD a 939 USD). Por el contrario, la cuota de mercado de medicamentos de primera línea del Servicio se redujo del 37,2 % al 19,2 % durante este tiempo, mientras que los precios aumentaron de 9,53 USD a 10,2 USD.

**Conclusión:**

El precio de los medicamentos contra la tuberculosis suministrados a través del Servicio fue generalmente inferior que en el mercado privado. Sin embargo, para alcanzar su potencial y satisfacer las necesidades del mayor número de pacientes de tuberculosis, el Servicio requiere una financiación pública más diversa y estable, así como mayor flexibilidad para participar en el mercado privado.

## Introduction

Tuberculosis remains a global public health concern. In 2013, there were an estimated 9 million incident cases worldwide, 480 000 of which involved multidrug-resistant tuberculosis.^1^ For tuberculosis as well as other conditions, disease control depends on more than the existence of curative treatment – it also depends on the drug supply, which is ultimately mediated by the pharmaceutical market.^2^^–^^7^ Consequently, disease control is profoundly influenced by the functioning of this market, particularly in resource-poor settings with a high disease burden. In addition, despite the existence of international quality-assurance standards, tuberculosis drugs are often either substandard or counterfeit.^8^^–^^10^ The use of substandard drugs reduces the chance of successful treatment and promotes the emergence of drug-resistance.^11^ Although the patents have expired on many tuberculosis drugs, the power of individual low-income countries with a high disease burden to negotiate cheaper treatment is limited. Second-line treatment for multidrug-resistant tuberculosis involves more protracted and complex chemotherapy and can cost a hundred times more than treating drug-sensitive tuberculosis.^12^^,^^13^

**Fig. 1:**
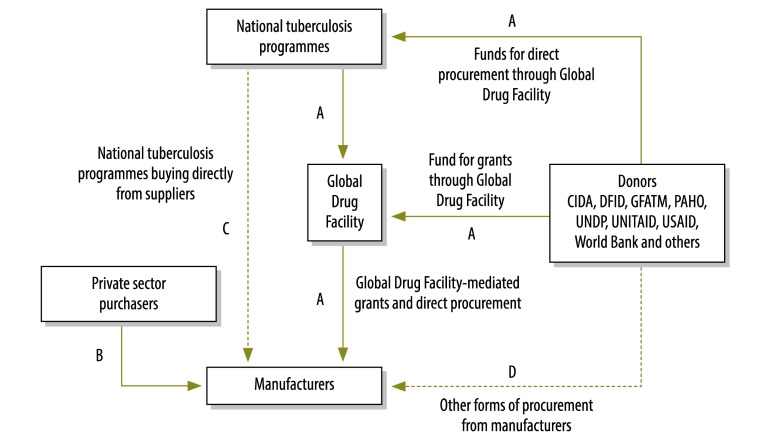
Principal funding flows^a^ in the tuberculosis drug market, 2001–2012

CIDA: Canadian International Development Agency; DFID: (United Kingdom) Department for International Development; GFATM: Global Fund to Fight AIDS, Tuberculosis and Malaria; PAHO: Pan American Health Organization; UNDP: United Nations Development Programme; USAID: United States Agency for International Development.

^a^ The solid lines indicate funding pathways for which data were available and which were included in the present analysis. The dashed lines indicate where data were lacking for drugs supplied outside the Global Drug Facility. The letters denote funding channels: A – national tuberculosis programmes buying through the Global Drug Facility; B – any client buying through the private market; C – national tuberculosis programmes buying directly from manufacturers; and D – other public sources of drugs.

In light of these issues, the Global Drug Facility was launched by the Stop TB Partnership in 2001 with the aim of using donor funding to consolidate demand from different countries and negotiate lower prices for quality-assured tuberculosis drugs.^14^^,^^15^ The facility now occupies a unique position in the global market for these drugs – in 2011, it supplied enough drugs to treat 35% of publicly notified cases of tuberculosis worldwide and an estimated 24% of all incident cases.^16^ However, the facility is only one participant in a complex, global tuberculosis drugs market (Fig. 1). Other drug purchasers include those in the private sector, national tuberculosis programmes and, in certain cases, donors themselves. In this environment, a defining feature of the Global Drug Facility model is the central role that international quality-assurance standards play in its operation: they are embedded in overall quality management so that stringent public procurement standards can be met.^17^ In the absence of such a framework, even manufacturers concerned about quality may find that the benefits of acquiring international quality-assurance certification do not necessarily outweigh the investments needed to meet these standards. By creating a large, stable market, a mechanism such as the Global Drug Facility provides clear incentives for a supply of drugs that meet international quality-assurance standards. In 2012, the value of this market for tuberculosis drugs exceeded 109 million United States dollars (US$).

Given that the Global Drug Facility plays such a large role in the tuberculosis drugs market, it is important to have some understanding of its influence on both sales volumes and drug prices. The aim of this study was to investigate changes in the price of the tuberculosis treatments supplied by the Global Drug Facility over the past 12 years of its operation and changes in its funding. In addition, we compared the price of tuberculosis treatment supplied by the Global Drug Facility with that of equivalent drugs purchased on the private market in 15 countries.

## Methods

The main funding flows in the global tuberculosis drug market are shown in Fig. 1, in which the solid lines indicate the flows for which data were available for our study. Details of the value of the funding channels labelled A in the figure were obtained from procurement data from the Global Drug Facility for the period 2001 to 2012. We derived the number of courses of treatment supplied from these data as described previously.^16^ In calculating drug prices, we incorporated the combination of drugs used in a full course of treatment for a single patient (Table 1). For second-line treatment, to cover a wide range of possible treatment regimens, we considered a cheaper, low-end regimen and a more expensive, high-end regimen (Table 1), as in previous work.^16^

**Table 1:** Drugs for tuberculosis, 2001–2012

Type and content of treatment	Quantity of drug in a dosage unit	Number of dosage units in one course of treatment^a^
**First-line **		
Four-drug fixed-dose combination; rifampicin, isoniazid, pyrazinamide, ethambutol	Rifampicin 150 mg, isoniazid 75 mg, pyrazinamide 400 mg and ethambutol 275 mg	168
Two-drug fixed-dose combination; rifampicin and isoniazid	Rifampicin 150 mg and isoniazid 75 mg	336
**Second-line (low-end)^b^**		
Kanamycin	1 g	180
Ethionamide	250 mg	2160
Cycloserine	250 mg	2160
Levofloxacin	250 mg	2160
**Second-line (high-end)^c^**		
Capreomycin	1 g	180
Protionamide	250 mg	2160
Cycloserine	250 mg	2160
Levofloxacin	250 mg	2160
4-Aminosalicylic acid	4 g	1440

^a^ Although 2011 treatment guidelines for multidrug-resistant tuberculosis increased the recommended treatment duration for injectable drugs from 6 to 8 months, orders delivered in 2012 had all been placed under previous guidelines. Accordingly, we assumed that all patients were treated for 6 months.

^b^ Low-end, second-line treatment regimens were those at the lower end of the price range for all possible regimens.

^c^ High-end, second-line treatment regimens were those at the more expensive end of the price range for all possible regimens.

We used Global Drug Facility data to calculate the cost of a single standard unit of treatment: (i) a fixed-dose combination pill for first-line treatment; and (ii) a pill or vial of injectable compound for second-line treatment. We then derived the cost of a course of treatment for an individual patient using the number of standard units required, as shown in Table 1. Generally we used the mean unit price for each drug and therefore the mean price of each treatment course but we also considered the price range by using the maximum and minimum unit prices for each drug. All prices are expressed in US$, the currency in which the Global Drug Facility purchases and supplies drugs.

In Fig. 1, funding channels B and C represent the private market. Data on these channels were obtained for 2002 to 2012 from IMS Health – an organization that collects information on drug purchases in a range of countries. Data from IMS Health covered 15 countries, including 10 with a high burden of tuberculosis and 11 with a high burden of multidrug-resistant tuberculosis (Table 2). These countries represented the range of support received from the Global Drug Facility: for example, India has been a major purchaser of drugs through the facility in recent years, whereas South Africa has had almost no involvement. We calculated the price of a treatment course as described above. To achieve consistency with Global Drug Facility data, we converted prices expressed in other currencies into US$ using the exchange rates in force at the time of each transaction.

**Table 2:** Countries in the IMS Health data set that received tuberculosis drugs from the private market, 2001–2012

Country	Tuberculosis notifications in the country since 2001 as a proportion of tuberculosis notifications globally (%)			Proportion of total Global Drug Facility supplies received by the country since 2001, by value (%)	
	Drug-sensitive tuberculosis^a^	Multidrug-resistant tuberculosis		First-line drugs	Second-line drugs
Bangladesh	2.35	0.38		6.28	1.03
Brazil	1.51	1.31		0.05	0.00
Bulgaria	0.05	0.13		0.00	0.17
China	15.64	2.22		0.00	6.00
Dominican Republic	0.08	0.10		0.16	0.52
India	24.91	7.24		31.01	17.39
Indonesia	4.51	0.42		5.9	0.81
Latvia	0.02	0.48		0.00	0.00
Lithuania	0.04	1.02		0.00	0.00
Pakistan	3.19	0.69		4.94	1.31
Peru	0.63	3.75		0.00	13.10
Philippines	2.75	1.62		3.72	5.20
Russian Federation	2.88	18.97		0.00	7.48
South Africa	5.88	19.27		0.00	0.05
Thailand	1.05	0.38		0.05	0.18
**Total**	**65.74**	**58.20**		**52.10**	**53.25**

**^a^** Since the rate of drug sensitivity testing was low worldwide, all patients who did not have a positive test result for drug-resistance were regarded as having drug-sensitive tuberculosis.

For this study, the private market included all sources of tuberculosis drugs that were not supplied by the Global Drug Facility or through any other international financing mechanism, irrespective of whether the drugs were purchased by public or private sector organizations (i.e. channels B and C in Fig. 1). We did not consider other public sources of drugs (i.e. channel D in Fig. 1) because of a lack of systematic price data. Since IMS Health data come from a variety of sources (e.g. retailers and hospitals), incorporate different taxes (e.g. sales and import taxes) and may include discounts for large purchase volumes, it was difficult to compare prices directly. Accordingly, we compared ex-works prices – that is, the prices of drugs purchased and collected at the site of their manufacture. For the private market, we used IMS Health estimates of ex-works prices; for drugs supplied by the Global Drug Facility, we used ex-works prices from facility purchasing data. It was not possible to quantify the uncertainty in IMS Health estimates of ex-works prices because relevant data were not available. To address this limitation, we estimated the magnitude of the price bias that would be needed to negate the findings of our analysis. We adjusted all prices for inflation in each country separately using data on consumer price indices from the World Bank. Then, to investigate global trends, we averaged prices across countries, weighted by the quantity of drugs supplied to each country.

Finally, for channel C in Fig. 1, it was not possible to compare countries, as it was for channel B, because of a lack of systematic, public data on the price of drugs procured by national tuberculosis programmes directly from manufacturers. One exception was South Africa, which has published procurement data for its tuberculosis programme.^18^ In this case, we were able to make a comparison with the Global Drug Facility’s prices.

## Results

**Fig. 2:**
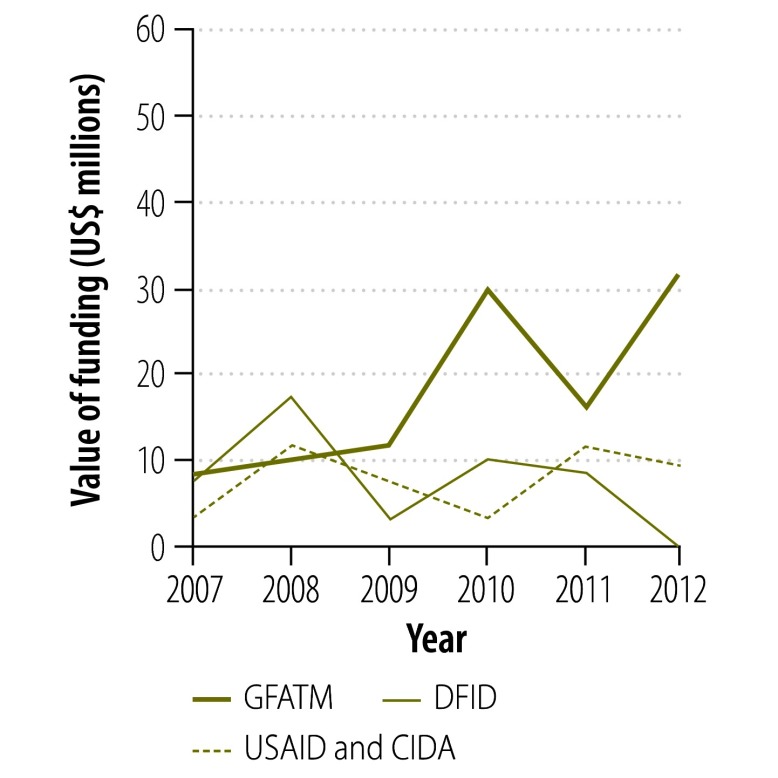
Funding sources^a,b^ for first-line tuberculosis drugs supplied through the Global Drug Facility, 2007–2012

CIDA: Canadian International Development Agency; DFID: (United Kingdom) Department for International Development; GFATM: Global Fund to Fight AIDS, Tuberculosis and Malaria; USAID: United States Agency for International Development; US$: United States dollars.

^a^ For clarity, only the three largest funding entities are shown.

^b^ Funding from the United States Agency for International Development and the Canadian International Development Agency was aggregated until 2010.

**Fig. 3:**
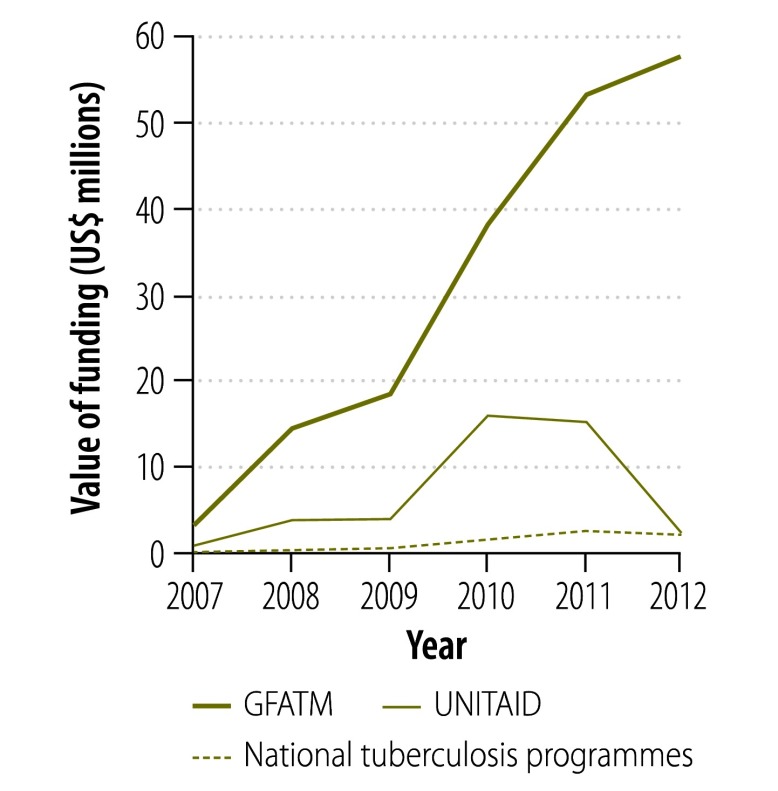
Funding sources^a^ for second-line tuberculosis drugs supplied through the Global Drug Facility, 2007–2012

GFATM: Global Fund to Fight AIDS, Tuberculosis and Malaria; US$: United States dollars.

^a^ For clarity, only the three largest funding entities are shown.

**Fig. 4:**
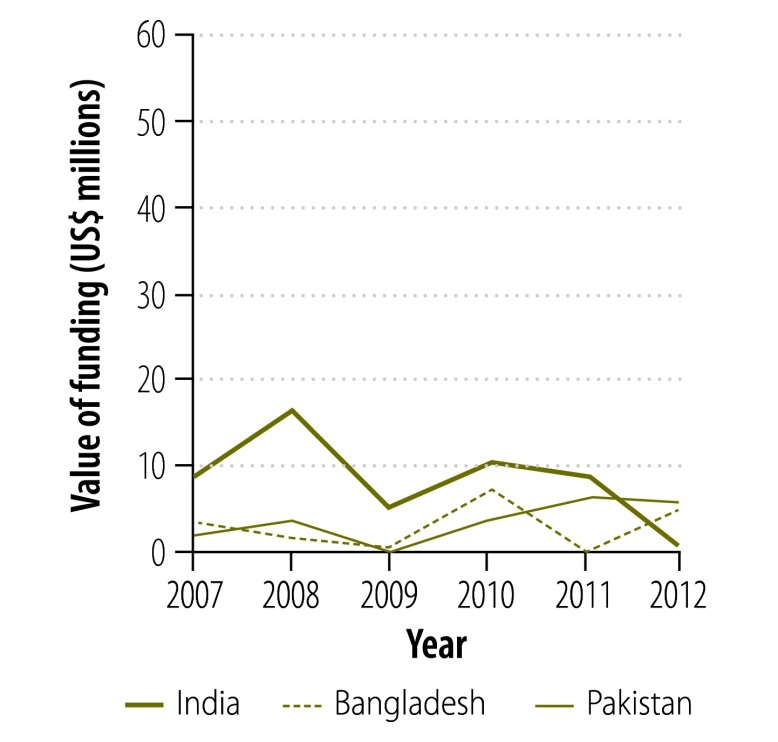
Funding to countries^a^ for first-line tuberculosis drugs from the Global Drug Facility, 2007–2012

**Fig. 5:**
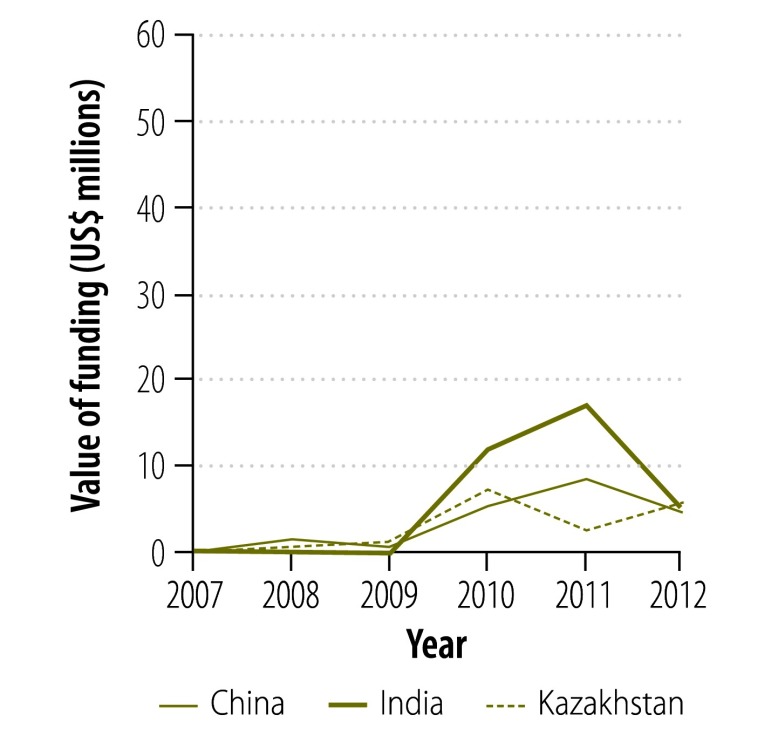
Funding to countries^a^ for second-line tuberculosis drugs from the Global Drug Facility, 2007–2012

**Fig. 6:**
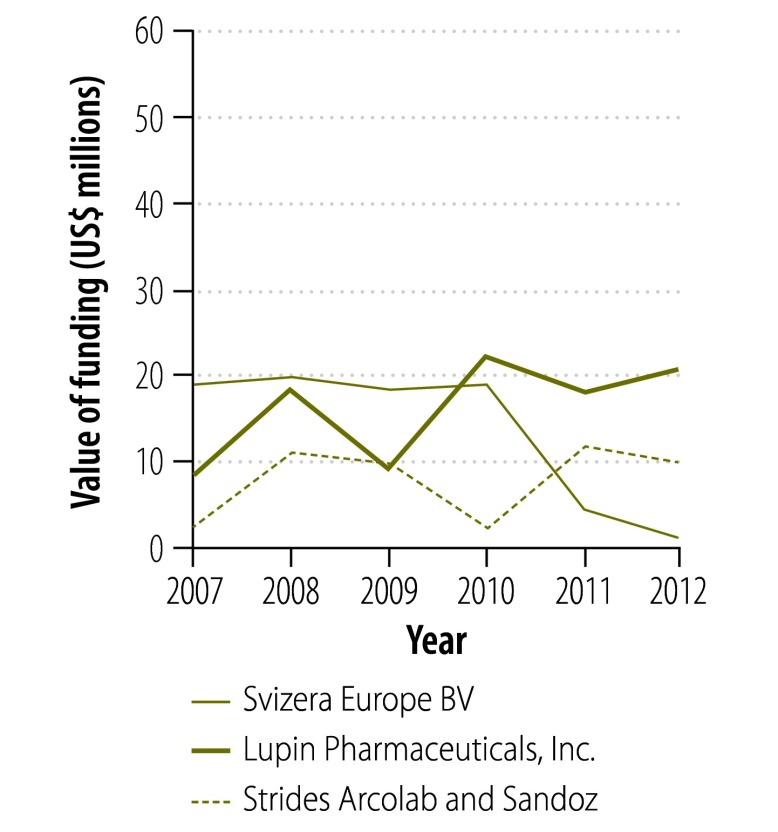
Funding flows to manufacturers^a^ of first-line tuberculosis drugs from the Global Drug Facility, 2007–2012

**Fig. 7:**
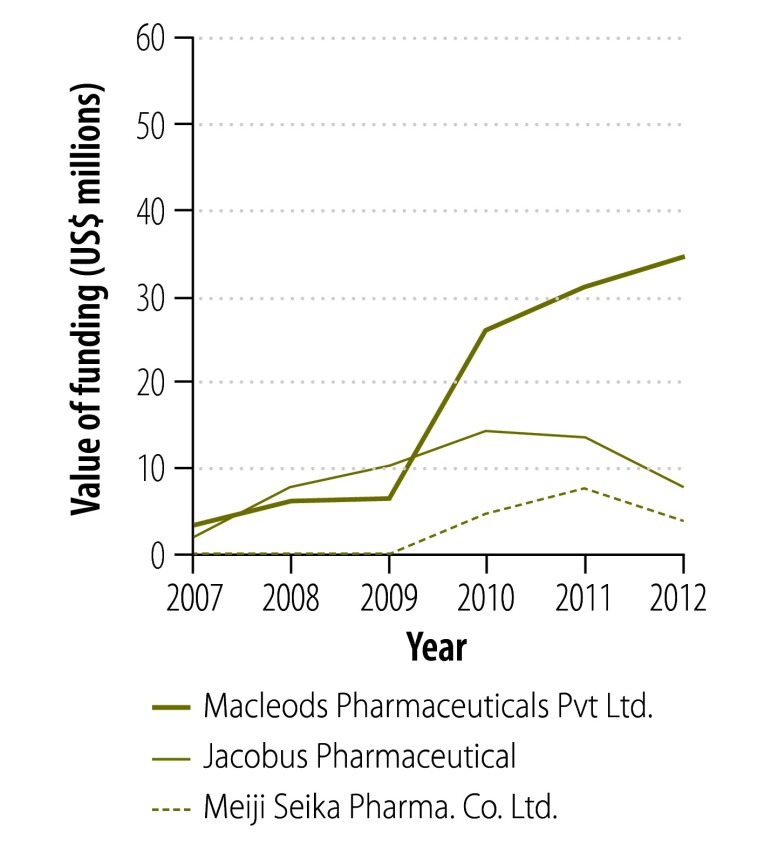
Funding flows to manufacturers^a^ of second-line tuberculosis drugs from the Global Drug Facility, 2007–2012

Fig. 2 and Fig. 3 show the change in donor involvement with the Global Drug Facility between 2007 and 2012 for first- and second-line tuberculosis drugs, respectively. Fig. 4, Fig. 5, Fig. 6 and Fig. 7 show the corresponding involvement of selected recipient countries and manufacturers with the facility. One key change in that period was a reduction in bilateral funding from the United Kingdom’s Department for International Development for first-line tuberculosis drugs in India. As a result, India stopped receiving these drugs through the Global Drug Facility. Overall, the proportion of the Global Drug Facility’s funding that came from the Global Fund to Fight AIDS, Tuberculosis and Malaria increased over time: in 2012, it was 73% (US$ 32.5 million of US$ 44.5 million) and 89% (US$ 57.8 million of US$ 65.2 million) for first- and second-line drugs, respectively. On the supply side, manufacturing remained highly concentrated: the largest three manufacturers together accounted for more than 67% by value of the first-line drugs supplied ($29.9 million of $44.5 million).

US$: United States dollars.

^a^ For clarity, only the three largest recipient countries are shown.

US$: United States dollars.

^a^ For clarity, only the three largest recipient countries are shown.

US$: United States dollars.

^a^ For clarity, only the three largest manufacturers are shown.

US$: United States dollars.

^a^ For clarity, only the three largest manufacturers are shown.

**Fig. 8:**
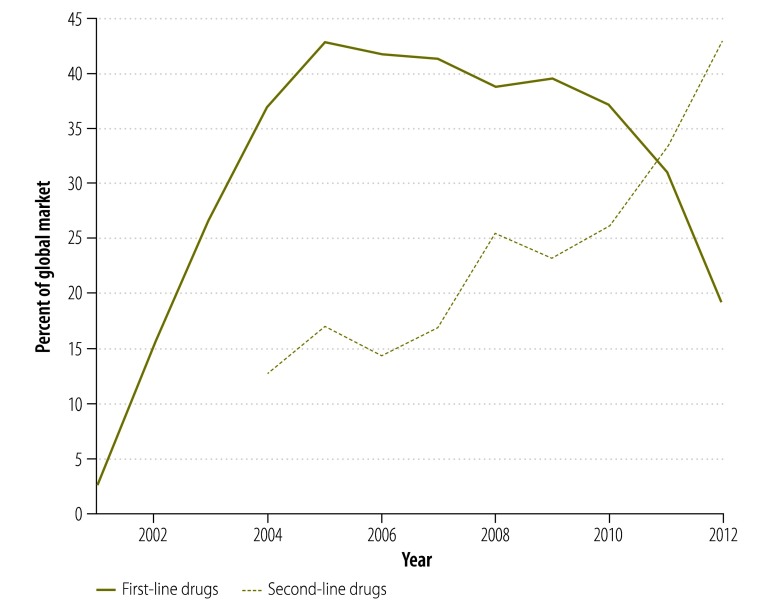
The Global Drug Facility’s share^a^ of the global market for tuberculosis drugs, 2001–2012

^a^ The estimated market share is the number of tuberculosis treatment courses supplied by the Global Drug Facility expressed as a percentage of the number of publicly notified cases of tuberculosis in the corresponding year.

Fig. 8 shows the change in the Global Drug Facility’s share of the market for first- and second-line tuberculosis drugs between 2001 and 2012. The graphs were derived by extending findings reported by Arinaminpathy et al.^16^ to 2012 and illustrate the number of treatment courses supplied each year by the Global Drug Facility as a percentage of the number of tuberculosis cases notified publicly in that year. Between 2010 and 2012, the Global Drug Facility’s market share of first-line drugs declined by 48% (from 37.2% to 19.2%). This decline was driven largely by the shifts in funding and demand illustrated in Fig. 2 and Fig. 4. In contrast, the Global Drug Facility’s market share of second-line drugs increased by 64% (from 26.1% to 42.9%) between 2010 and 2012.

### Drug price dynamics

**Fig. 9:**
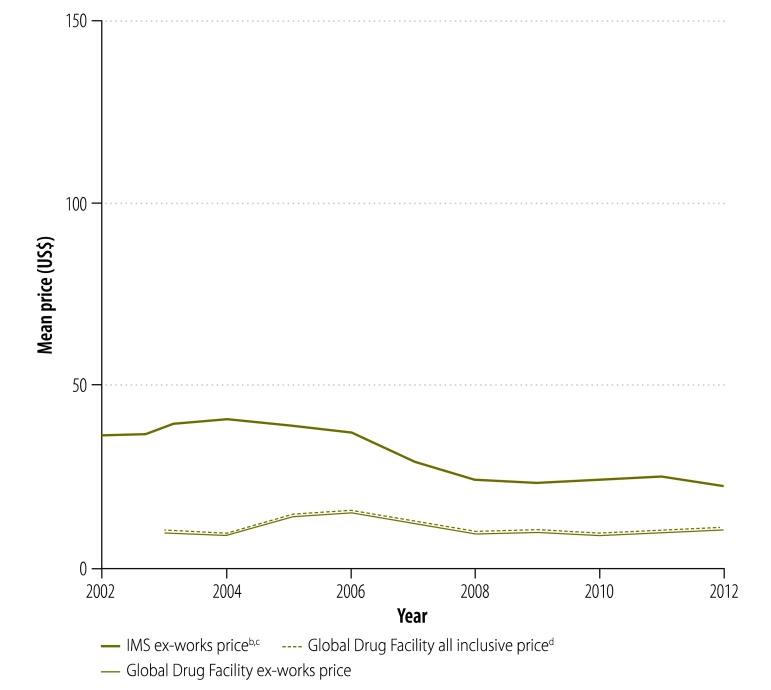
Price^a^ of a course of first-line tuberculosis drugs, 2002–2012

**Fig. 10:**
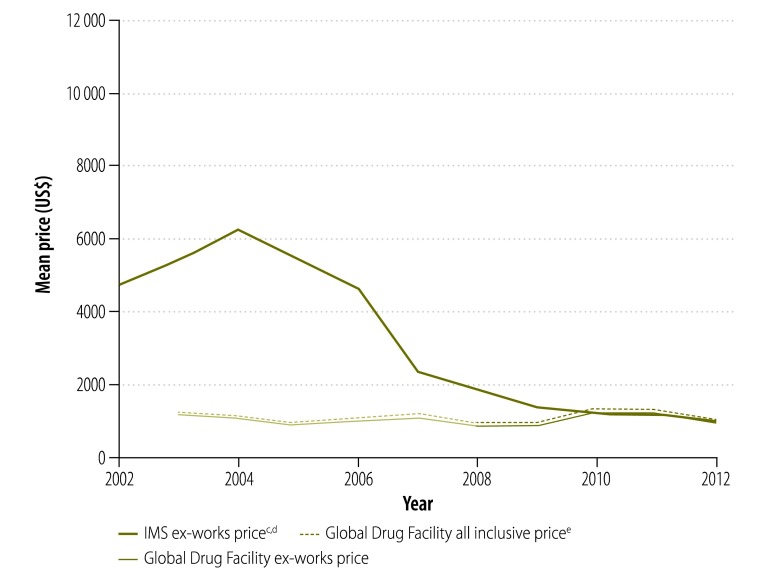
Price^a^ of a course of low-end, second-line^b^ tuberculosis drugs, 2002–2012

**Fig. 11:**
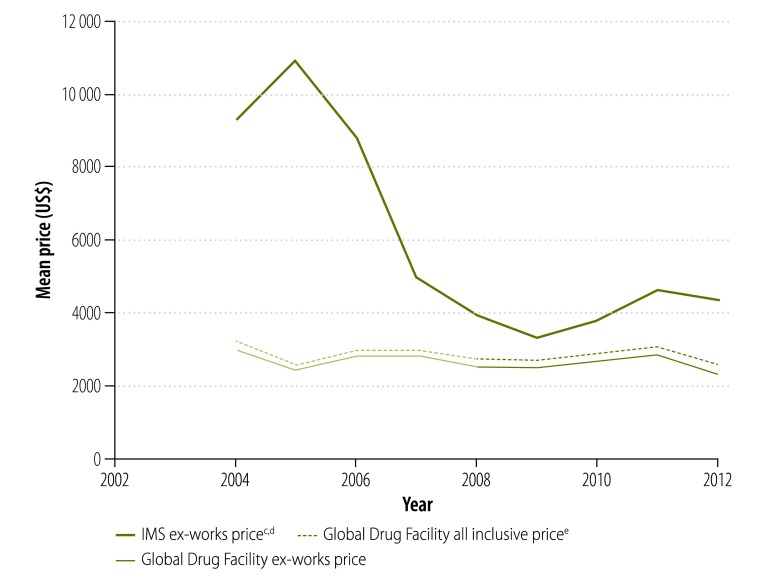
Price^a^ of a course of high-end, second-line^b^ tuberculosis drugs, 2002–2012^c^

**Fig. 12:**
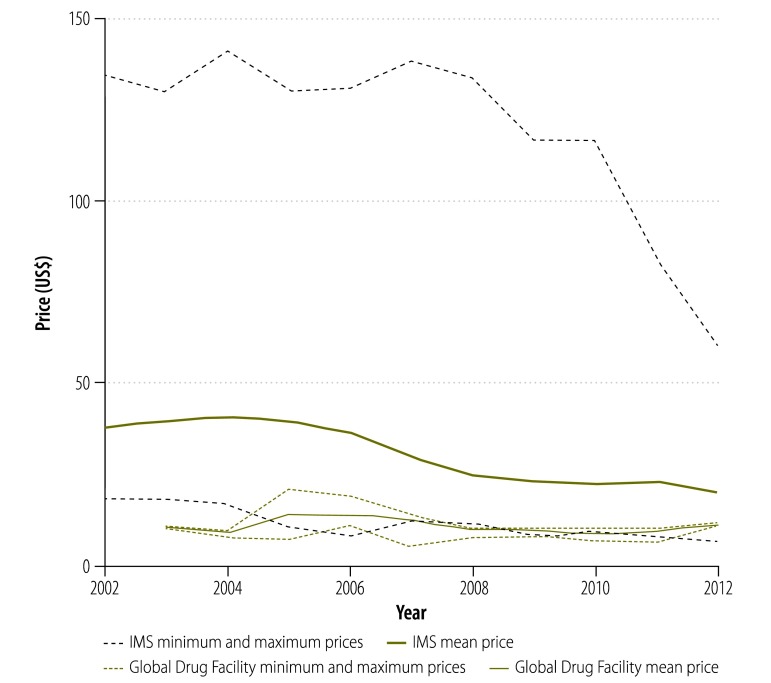
Price^a^ range of a course of first-line tuberculosis drugs, 2002–2012

In our analysis, we looked at the prices paid for treatment by national tuberculosis programmes supplied by the Global Drug Facility rather than the bid prices initially put forward by manufacturers. Fig. 9 shows that, since 2001, the price of a course of treatment with first-line drugs per patient was less for drugs supplied through the Global Drug Facility than through the private market. In 2003, the price was 71% lower (US$ 10.9 versus US$ 37.8) and, in 2012, it was 53% (US$ 10.2 versus US$ 22.1) lower. However, the price increased by 7% (from US$ 9.53 to US$ 10.2) between 2010 and 2012. Similarly, in 2004, the price of a course of treatment with low-end, second-line drugs was 82% lower (US$ 1066 versus US$ 5724) through the Global Drug Facility than the private market (Fig. 10) and the price of treatment with high-end regimens was 65% lower (US$ 3117 versus US$ 8930; Fig. 11). However, the disparity narrowed over the years as the private sector reduced its prices. Between 2010 and 2012, the price of second-line drugs supplied by the Global Drug Facility decreased by 24% (from US$ 1231 to US$ 939) and 16% (from US$ 2843 to US$ 2393) for low-end and high-end regimens, respectively. When we estimated the price bias that would be necessary for true prices in the private market to be 85% of Global Drug Facility prices or lower, we found that the potential bias for first-line drugs in 2012 would have had to exceed 155% of true private market prices (a bias of US$ 22.13, over hypothetical true market prices of US$ 8.68). Similarly, for second-line drugs, the bias in 2012 would have had to exceed 14% (US$ 911 versus US$ 798) and 105% (US$ 4178 versus US$ 2034) for low-end and high-end regimens, respectively. In addition to the mean prices shown in Fig. 9, Fig. 12 shows minimum and maximum prices globally between 2002 and 2012. As might be expected, given that a central purchasing entity was being compared with a diverse private market, the variation in Global Drug Facility prices was markedly less than the variation in private market prices.

US$: United States dollars.

^a^ Prices were adjusted for inflation in the country of sale.

^b^ IMS Health data were aggregated over 15 countries.

^c^ The ex-works price is the price of a drug purchased and collected at the site of its manufacture.

^d^ The inclusive price included an estimate of additional procurement and logistic costs associated with, for example, transportation, procurement agents’ commissions, insurance and quality control.

US$: United States dollars.

^a^ Prices were adjusted for inflation in the country of sale.

^b^ Low-end, second-line treatment regimens were those at the lower end of the price range for all possible regimens.

^c^ IMS Health data were aggregated over 15 countries.

^d^ The ex-works price is the price of a drug purchased and collected at the site of its manufacture.

^e^ The inclusive price included an estimate of additional procurement and logistic costs associated with, for example, transportation, procurement agents’ commissions, insurance and quality control.

Note: regular Global Drug Facility supplies were only established from 2008 onwards. Accordingly, Global Drug Facility prices before this year are shown in a lighter shade.

US$: United States dollars.

^a^ Prices were adjusted for inflation in the country of sale.

^b^ High-end, second-line treatment regimens were those at the higher end of the price range for all possible regimens.

^c^ IMS Health data were aggregated over 15 countries.

^d^ The ex-works price is the price of a drug purchased and collected at the site of its manufacture.

^e^ The inclusive price included an estimate of additional procurement and logistic costs associated with, for example, transportation, procurement agents’ commissions, insurance and quality control.

Note: regular Global Drug Facility supplies were only established from 2008 onwards. Accordingly, Global Drug Facility prices before this year are shown in a lighter shade

US$: United States dollars.

^a^ Prices were adjusted for inflation in the country of sale.

**Fig. 13:**
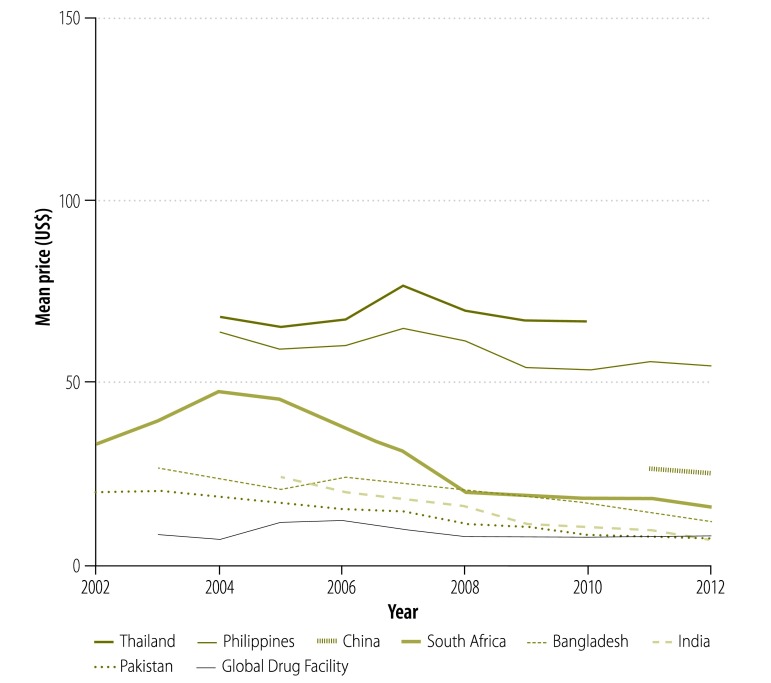
Price^a^ of a course of first-line tuberculosis drugs, by country,^b^ 2002–2012

**Fig. 14:**
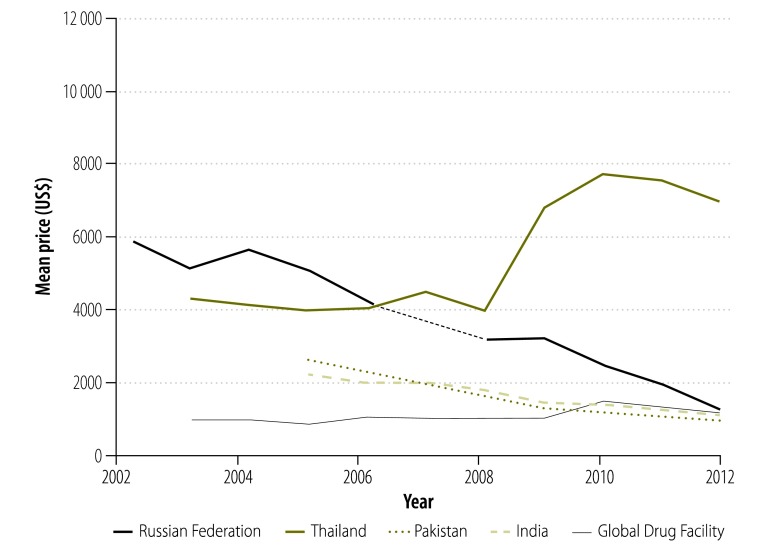
Price^a^ of a course of low-end,^b^ second-line tuberculosis drugs, by country,^c,d^ 2002–2012

**Fig. 15:**
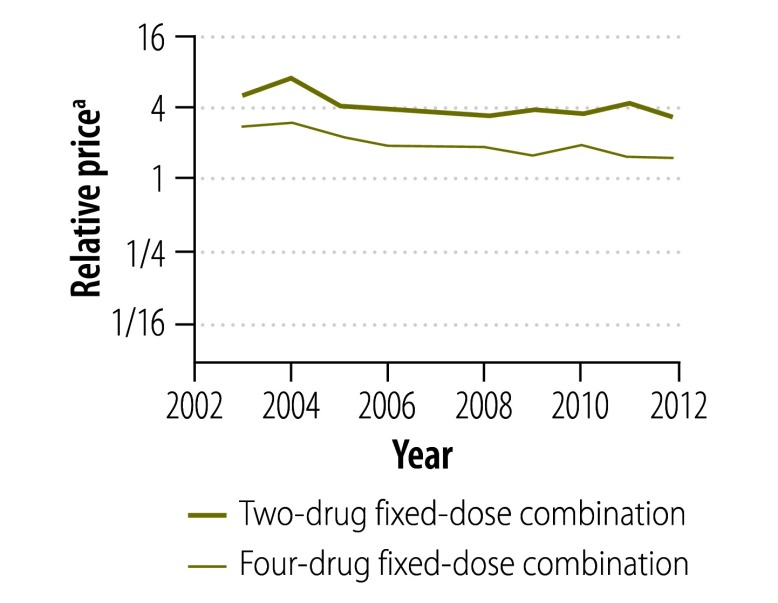
Relative price^a^ of a course of first-line tuberculosis treatment, by treatment, 2002–2012

^a^ The relative price was the ratio of the mean global price of the treatment on the private market to that of the treatment supplied by the Global Drug Facility. Where this is 1, the two prices were equal.

**Fig. 16:**
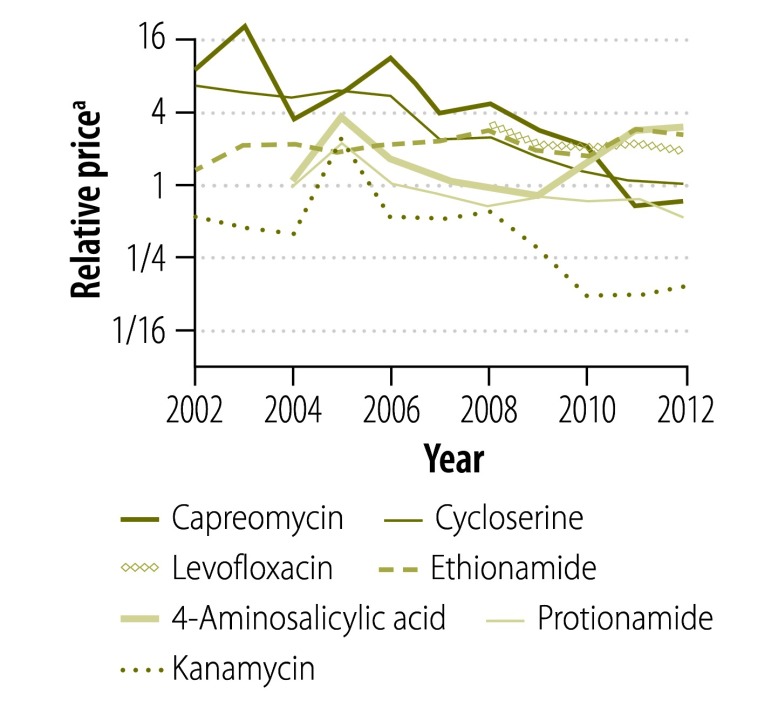
Relative price^a^ of a course of second-line tuberculosis treatment, by drug, 2002–2012

^a^ The relative price was the ratio of the mean global price of the drug on the private market to that of the drug supplied by the Global Drug Facility. Where this is 1, the two prices were equal.

Fig. 13 and Fig. 14 illustrate the variation between 2002 and 2012 in the price of a course of treatment with first- and second-line drugs, respectively, in selected countries. It shows that the price of drugs supplied by the Global Drug Facility was less than that of drugs available in the private market for all countries. Fig. 15 and Fig. 16 (both available at: http://www.who.int/bulletin/volumes/93/4/14-147256) display the price of individual first- and second-line treatments, respectively, obtained through the Global Drug Facility relative to that of treatment purchased from the private market between 2002 and 2012. The price of most drugs was consistently higher when purchased from the private market. The exceptions were protionamide, capreomycin and kanamycin – their mean price on the private market was 33% (US$ 0.020 versus US$ 0.062), 44% (US$ 1.24 versus US$ 2.84) and 11% (US$ 0.10 versus US$ 0.97) respectively, of the corresponding price from the Global Drug Facility. Nonetheless, since kanamycin accounts for only around 20% (US$ 189 of US$ 939), of the price of a course of low-end, second-line treatment from the Global Drug Facility the overall price of treatment was still lower than it would have been on the private market.

US$: United States dollars.

^a^ Prices were adjusted for inflation in the country of sale.

^b^ Only countries for which data were available on all drugs required for a full treatment course were included.

US$: United States dollars.

^a^ Prices were adjusted for inflation in the country of sale.

^b^ Low-end, second-line treatment regimens were those at the lower end of the price range for all possible regimens.

^c^ Only countries for which data were available on all drugs required for a full treatment course were included.

^d^ The dashed black line shows interpolated unavailable data for the Russian Federation for 2007.

**Fig. 17:**
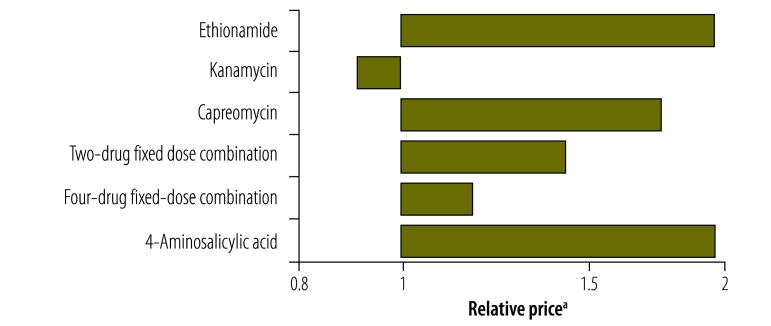
Relative price^a^ of a course of tuberculosis treatment purchased by South Africa, by treatment, 2012

^a^ The relative price was the ratio of the mean price of the treatment when procured directly from manufacturers by the South African national tuberculosis programme to the price of the treatment supplied by the Global Drug Facility.

Fig. 17 shows the ratio of the price of tuberculosis drugs procured directly from manufacturers by the national tuberculosis programme in South Africa to the price of drugs from the Global Drug Facility. Again the figure illustrates that, with the exception of kanamycin, the price of drugs supplied by the Global Drug Facility was lower than that of drugs obtained directly from private markets. Moreover, it should be noted that, although manufacturers supplied the Global Drug Facility with drugs that met international quality-assurance standards, many had different production lines that were used to supply other clients, including national programmes. Overall therefore, drugs, including kanamycin, that were supplied by sources other than the Global Drug Facility were of uncertain quality, whether or not they were provided by manufacturers who also supplied the facility.

## Discussion

Our analysis suggests that a mechanism such as the Global Drug Facility can indeed secure lower prices for drugs that meet international quality-assurance standards than are available for unregulated drugs of unknown quality on the private market. Moreover, the Global Drug Facility’s prices varied considerably less than those in the private market. This could greatly assist planning, both for countries procuring drugs and for manufacturers, who would be able to anticipate future demand. In this way, mechanisms such as the Global Drug Facility could create and support identifiable, transparent markets for internationally quality-assured drugs. Nonetheless, the Global Drug Facility’s success in reducing prices was not universal: some second-line drugs, particularly kanamycin, cost substantially more from the Global Drug Facility than equivalent drugs of unknown quality offered on the private market. A key factor in the price of kanamycin was the limited availability of its active pharmaceutical ingredient – only a few suppliers met stringent World Health Organization quality criteria. Future interventions in the global dug market should address factors limiting the drug supply.

In addition, our analysis highlights the risks to any initiative based on consolidating demand such as the Global Drug Facility. For example, the facility’s operations were affected by recent changes in funding. How might such risks be mitigated? First, the health of the market for internationally quality-assured drugs depends on its size: a larger market can accommodate more manufacturers and promote competition as well as offering greater scope for economies of scale that will further reduce drug prices. It is, therefore, important to reverse the loss in the sales volume of first-line drugs we observed recently. Currently the Global Drug Facility supplies only the public sector (i.e. national tuberculosis programmes). However, the role of the private sector in controlling tuberculosis is being increasingly recognized and there may be new opportunities for the facility to supply internationally quality-assured drugs outside the public sector, where they are also needed.^19^^–^^23^ Second, in addition to its current model of inviting applications for support from individual countries, the Global Drug Facility could also become a strong competitor if, in certain cases, it participated directly in national tenders (i.e. without a procurement agent) and became one supplier among many bidding to provide drugs for national tuberculosis programmes. If the Global Drug Facility received money from these programmes themselves, its reliance on donor support would be reduced. The large national tuberculosis programmes in India and South Africa could be important in this regard.

On the donor side, our results highlight the risks of unstable funding sources and of funding coming from an increasingly small number of donors. However, it is important to note that donors have an influence that goes beyond their effect on purchasing power. For example, donor support encourages national tuberculosis programmes to adopt international guidelines (this is often a condition of support), ensures there is a pool of prequalified manufacturers who produce internationally quality-assured drugs and enables the Global Drug Facility to charge the lowest possible fees to participating countries, thus keeping costs low. Consequently, in the future, the Global Drug Facility should continue to serve public markets as it does at present, while at the same time seeking ways to relax constraints on the supply of tuberculosis drugs so that the facility can compete more directly in the tuberculosis drug market than it does at present. This combined approach could dramatically increase the level of demand managed by the Global Drug Facility, provide it with greater leverage and enable it to stimulate and sustain the market.

Our analysis has several limitations. The lack of fine-grained, country-specific data from both IMS Health and the Global Drug Facility meant that we had to compare prices at the ex-works level rather than the patient level. Further, there may have been inaccuracies in IMS estimates of ex-works prices. However, if prices were underestimated, our finding that the Global Drug Facility negotiated prices that were lower than, or comparable to, those in the private market would be strengthened. However, if prices were overestimated, our analysis suggests that the error would have had to be very large to negate our qualitative findings.

Our work suggests areas for future study. For example, apart from some research carried out in specific contexts,^10^^,^^20^ few systematic, longitudinal studies have investigated the quality and quantity of drugs supplied outside the Global Drug Facility – that is, the funding channels represented by dashed lines in Fig. 1. There is, then, a need for systematic, large-scale surveys of the price, volume and quality of drugs available in different countries. This could be achieved by establishing national observatories. Such data would be invaluable for building a comprehensive picture of the most cost-effective sources of tuberculosis drugs.

In conclusion, our analysis throws light on how the Global Drug Facility’s operations since 2001 have influenced the dynamics of the market for internationally quality-assured tuberculosis drugs. Although challenging, it is essential that the global health community fully engages with such complex, global markets. The lessons learnt from the operation of the Global Drug Facility and other similar interventions will be invaluable in future discussions about the role of such models of engagement, to the common benefit of donors, governments and patients.

## References

[1] Global Tuberculosis Report. Geneva: World Health Organization; 2014. pp.1–171. Available from: http://www.who.int/tb/publications/global_report/en/ [cited 2015 Feb 17].

[2] Nunn AS, Fonseca EM, Bastos FI, Gruskin S, Salomon JA. Evolution of antiretroviral drug costs in Brazil in the context of free and universal access to AIDS treatment. PLoS Med. 2007 11 13;4(11):e305. 10.1371/journal.pmed.0040305 18001145 PMC2071936

[3] Untangling the web of antiretroviral price reductions: 17th edition – July 2014. Geneva: Médecins Sans Frontières; 2014. pp.1–100. Available from: http://www.msfaccess.org/content/untangling-web-antiretroviral-price-reductions-17th-edition-–-july-2014 [cited 2015 Feb 17].

[4] UNITAID 5 year evaluation. Summary. Geneva: UNITAID; 2012. Available from: http://www.unitaid.eu/images/Five-year-evaluation/5YE%20Exec%20Summary-UNITAID%202012-12-03%2016h00.pdf[cited 2014 Jan 18].

[5] Bermudez J, ’t Hoen E. The UNITAID patent pool initiative: bringing patents together for the common good. Open AIDS J. 2010;4(1):37–40. 10.2174/1874120701004010037 20309404 PMC2842943

[6] Bärnighausen T, Kyle M, Salomon JA, Waning B. Assessing the population health impact of market interventions to improve access to antiretroviral treatment. Health Policy Plan. 2012 9;27(6):467–76. 10.1093/heapol/czr058 21914713 PMC3431498

[7] ’t Hoen EFM, Hogerzeil HV, Quick JD, Sillo HB. A quiet revolution in global public health: the World Health Organization’s prequalification of medicines programme. J Public Health Policy. 2014 5;35(2):137–61. 10.1057/jphp.2013.53 24430804

[8] Bate R, Tren R, Mooney L, Hess K, Mitra B, Debroy B, et al. Pilot study of essential drug quality in two major cities in India. PLoS ONE. 2009;4(6):e6003. 10.1371/journal.pone.0006003 19547757 PMC2695555

[9] Bate R, Jensen P, Hess K, Mooney L, Milligan J. Substandard and falsified anti-tuberculosis drugs: a preliminary field analysis. Int J Tuberc Lung Dis. 2013 3;17(3):308–11. 10.5588/ijtld.12.0355 23321423

[10] Survey of the quality of anti-tuberculosis drugs circulating in selected newly independent states of the former Soviet Union. Geneva: World Health Organization; 2011. Available from: http://apps.who.int/medicinedocs/documents/s19053en/s19053en.pdf?ua=1 [cited 2015 Jan 13].

[11] Caminero JA. Multidrug-resistant tuberculosis: epidemiology, risk factors and case finding. Int J Tuberc Lung Dis. 2010 4;14(4):382–90. 20202293

[12] Caminero JA, Sotgiu G, Zumla A, Migliori GB. Best drug treatment for multidrug-resistant and extensively drug-resistant tuberculosis. Lancet Infect Dis. 2010 9;10(9):621–9. 10.1016/S1473-3099(10)70139-0 20797644

[13] Ahuja SD, Ashkin D, Avendano M, Banerjee R, Bauer M, Bayona JN, et al.; Collaborative Group for Meta-Analysis of Individual Patient Data in MDR-TB. Multidrug resistant pulmonary tuberculosis treatment regimens and patient outcomes: an individual patient data meta-analysis of 9,153 patients. PLoS Med. 2012;9(8):e1001300. 10.1371/journal.pmed.1001300 22952439 PMC3429397

[14] Kumaresan J, Smith I, Arnold V, Evans P. The Global TB Drug Facility: innovative global procurement. Int J Tuberc Lung Dis. 2004 1;8(1):130–8. 14974756

[15] Matiru R, Ryan T. The Global Drug Facility: a unique, holistic and pioneering approach to drug procurement and management. Bull World Health Organ. 2007 5;85(5):348–53. 10.2471/BLT.06.035402 17639218 PMC2636664

[16] Arinaminpathy N, Cordier-Lassalle T, Vijay A, Dye C. The Global Drug Facility and its role in the market for tuberculosis drugs. Lancet. 2013 10 19;382(9901):1373–9. 10.1016/S0140-6736(13)60896-X 23726162

[17] Global Drug Facility TB Programme. Quality assurance policy and procedures. Geneva: Global TB Drug Facility & Stop TB Partnership, World Health Organization; 2010. Available from: http://www.stoptb.org/assets/documents/gdf/drugsupply/GDF%20QA%20Policy%20and%20Procedures.pdf [cited 2014 Feb 9].

[18] Supply and delivery of anti-tuberculosis medicines to the Department of Health for the period 1 August 2013 to 31 July 2015. Pretoria: Republic of South Africa Department of Health; 2013. pp. 1–28. Available from: http://www.health.gov.za/docs/contructs/HP01-2013CoCircular.pdf [cited 2015 Feb 17].

[19] Khan AJ, Khowaja S, Khan FS, Qazi F, Lotia I, Habib A, et al. Engaging the private sector to increase tuberculosis case detection: an impact evaluation study. Lancet Infect Dis. 2012 8;12(8):608–16. 10.1016/S1473-3099(12)70116-0 22704778

[20] Wells WA, Ge CF, Patel N, Oh T, Gardiner E, Kimerling ME. Size and usage patterns of private TB drug markets in the high burden countries. PLoS One. 2011;6(5):e18964. 10.1371/journal.pone.0018964 21573227 PMC3087727

[21] Laserson KF, Kenyon AS, Kenyon TA, Layloff T, Binkin NJ. Substandard tuberculosis drugs on the global market and their simple detection. Int J Tuberc Lung Dis. 2001 5;5(5):448–54. 11336276

[22] Hazarika I. Role of private sector in providing tuberculosis care: evidence from a population-based survey in India. J Glob Infect Dis. 2011 1;3(1):19–24. 10.4103/0974-777X.77291 21572604 PMC3068573

[23] Hoa NB, Cobelens FGJ, Sy DN, Nhung NV, Borgdorff MW, Tiemersma EW. Diagnosis and treatment of tuberculosis in the private sector, Vietnam. Emerg Infect Dis. 2011 3;17(3):562–4. 10.3201/eid1703.101468 21392464 PMC3166026

